# MicroRNAs in Intervertebral Disc Degeneration, Apoptosis, Inflammation, and Mechanobiology

**DOI:** 10.3390/ijms21103601

**Published:** 2020-05-20

**Authors:** Petra Cazzanelli, Karin Wuertz-Kozak

**Affiliations:** 1 Department of Biomedical Engineering, Rochester Institute of Technology (RIT), Rochester, NY 14623, USA; pc1124@rit.edu; 2 Schön Clinic Munich Harlaching, Spine Center, Academic Teaching Hospital and Spine Research Institute of the Paracelsus Medical University Salzburg (Austria), 81547 Munich, Germany

**Keywords:** miRNA, degenerative disc disease, ECM, MMP, senescence, nucleus pulposus, annulus fibrosus, cartilaginous endplate

## Abstract

Intervertebral disc (IVD) degeneration is a multifactorial pathological process associated with low back pain, the leading cause of years lived in disability worldwide. Key characteristics of the pathological changes connected with degenerative disc disease (DDD) are the degradation of the extracellular matrix (ECM), apoptosis and senescence, as well as inflammation. The impact of nonphysiological mechanical stresses on IVD degeneration and inflammation, the mechanisms of mechanotransduction, and the role of mechanosensitive miRNAs are of increasing interest. As post-transcriptional regulators, miRNAs are known to affect the expression of 30% of protein-coding genes and numerous intracellular processes. The dysregulation of miRNAs is therefore associated with various pathologies, including degenerative diseases such as DDD. This review aims to give an overview of the current status of miRNA research in degenerative disc pathology, with a special focus on the involvement of miRNAs in ECM degradation, apoptosis, and inflammation, as well as mechanobiology.

## 1. Introduction

Disc degeneration is a pathological process that leads to the deterioration of intervertebral discs (IVDs), the connective tissue between vertebrae which plays a crucial role in spinal kinematics. The degenerative processes occur on the tissue, cellular, and molecular level, leading to major changes in the morphology and physiology of the disc, and ultimately resulting in its decreased capability to bear compressive loads. Being a multifactorial disease, its etiology is as of yet still not completely understood. However, it is widely recognized that several factors, such as genetic predisposition, age, lifestyle (obesity, smoking, depressive symptoms), and nonphysiological mechanical loading contribute to its progression [1,2,3]. Beyond that, IVD degeneration is known to be associated with low back pain (LBP) [4]. This is of significance as LBP has been confirmed as the leading cause of years lived in disability worldwide in recent decades, affecting 80% of adults at some point in their lives [5]. Direct costs associated with LBP are estimated to run as high as $90 billion per year in the United States alone [6]. Taking into account the indirect costs caused by disability, such as the reduced productivity of patients, the overall socio-economic implications of LBP are posing a notable challenge for societies [7]. 

Several pathological changes in the IVD are connected with disc degeneration, amongst which degradation of the extracellular matrix, inflammation, and cell loss (apoptosis) are the most prevalent [1]. Focusing on the extracellular matrix (ECM), which plays an essential role in the mechanical functionality of the IVD, two main components are vital for its integrity: the type I and type II collagen network that provides tensile strength [8], and water-binding proteoglycans such as aggrecan [9]. However, metabolic dysregulation of *Nucleus pulposus* (NP) cells (i.e., cells in the central region of the IVD) results in their reduced ability to synthesize these ECM components while increasingly secreting ECM degradative molecules such as matrix metalloproteinases (MMP) and a disintegrin and metalloproteinases with thrombospondin motifs (ADAMTS) [10,11]. In consequence, dysregulation combined with proteoglycan breakdown leads to a diminished water-binding capacity of the tissue and finally to its structural collapse. Although the degenerative processes first arise in the NP, it later involves the *Annulus fibrosus* (AF) (i.e., the outer zone of the IVD), with the boundary between both tissues ultimately being lost [2,12,13,14]. ECM degradation is further enhanced by cell loss, a well-studied contributor to disc degeneration. Cell loss can be caused by programmed cell death (apoptosis) and is accompanied by cell senescence, with underlying factors such as mechanical stresses inducing both mechanisms [15,16,17].

Apart from ECM degradation and cell loss, inflammation also plays an important role and has emerged as a distinguishing factor between asymptomatic disc degeneration and symptomatic disc degeneration, often termed degenerative disc disease (DDD) [18,19]. It is well known that NP cells increasingly release a number of proinflammatory cytokines with progressive degrees of degeneration and often pain development, with tumor necrosis factor α (TNFα), interleukin (IL)-1β, IL-6, and IL-17 being the most prominent. These cytokines have been proved to promote matrix degradation and to activate a host immune response, eventually leading to the infiltration of immune cells and nerve fibers. The latter is particularly relevant because nerve infiltration is the source of pain associated with DDD [20]. 

ECM degradation, apoptosis, and inflammation are termed as the hallmarks of DDD and are known to be interconnected and interdependent from each other [2]. Proinflammatory cytokines contribute to the dysregulation of the ECM metabolism by upregulating the expression of ECM degradative enzymes and downregulating ECM structural components [18,20]. The inherent degradation of ECM leads to an extracellular accumulation of ECM fragments, which further stimulate the inflammatory response of NP cells [21]. Additionally, higher rates of apoptosis and senescence in IVD tissue are connected with lower ECM production capabilities and inflammation, the latter caused by the senescent-associated secretory phenotype [15,22,23].

In recent years, the interplay of mechanics and biology, termed mechanobiology, as well as its role in IVD degeneration, has been of growing interest. Nonphysiological mechanical loading of the IVD has been shown to be tightly associated with matrix degradation and changes in cellular physiology [24,25,26,27,28]. Several attempts have been made to explain the interdependency of mechanobiology, ECM degradation, cell loss, and inflammation, as well as possible feedback loops [12,29,30]. However, the observed connection between these mechanisms is not yet sufficiently understood and adds to the multifactorial nature of pathological changes associated with IVD degeneration.

Based on the current knowledge and understanding of IVD degeneration, standard therapy has been relying on pharmacological treatments, physiotherapy and, as a last resort, invasive surgical procedures, such as spine fusion or arthroplasty [31,32]. Due to the major limitations of these treatments, including modest success rates, invasiveness, and high costs, there has been a high demand for novel, targeted treatments that counteract the degenerative processes and reduce pain. Several approaches are being studied and tested as possible treatment options, amongst which cell therapies, endogenous repair strategies by activation of IVD reparative cells, and treatment based on biological factors such as microRNAs (miRNAs) are the most promising [33,34].

As key regulators of gene expression, miRNAs, a type of small noncoding RNAs, inhibit the translational process by binding to the 3′-untranslated region (3′-UTR) of target mRNA molecules, often leading to their degradation. In general, primary miRNAs are generated in the nucleus and further processed to mature miRNAs in the cytoplasm by the enzyme Dicer. After incorporation into the RNA-induced silencing complex (RISC), they can target and inhibit the translation of multiple mRNAs, thereby regulating approximately 30% of human protein-coding genes and multiple intracellular processes, including cell proliferation, apoptosis, and cytokine release [35]. Furthermore, miRNAs are known to interact with other endogenous RNAs, such as long noncoding RNAs, circular RNAs, and mRNAs, forming an extensive network of gene regulation in IVD cells, which was recently described by Zhu et al. [36]. Dysregulation of miRNAs has been associated with several pathological conditions, including cancer [37], cardiovascular diseases [38], as well as osteoarthritis [39] and IVD degeneration [40]. This has fueled the interest in miRNAs, their role in DDD, and their potential as novel biomarkers and therapeutics. 

**Figure 1:**
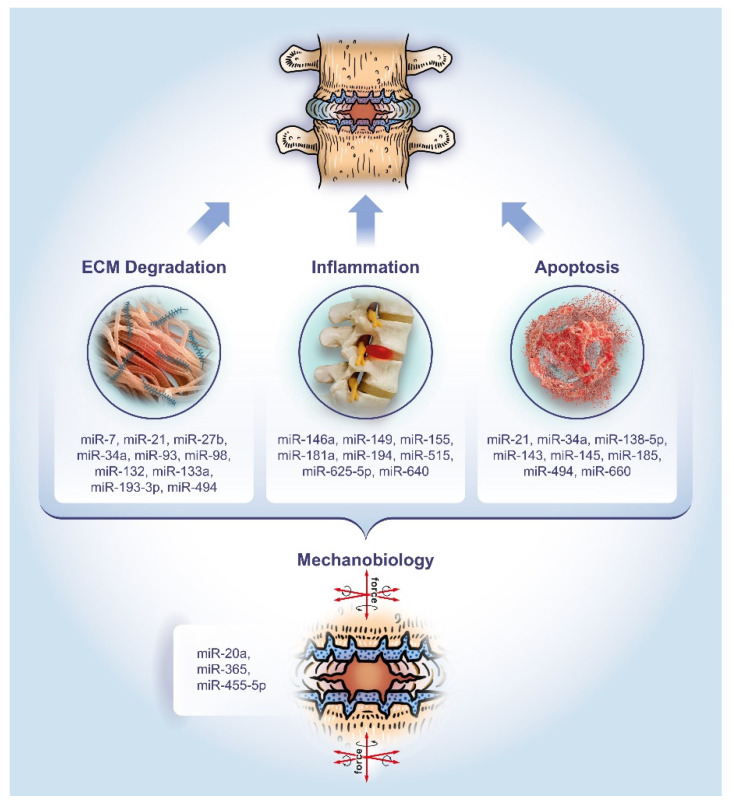
The role of microRNAs in the pathology of DDD. The miRNAs listed have been studied for their involvement in extracellular matrix (ECM) degradation, apoptosis, and inflammation, as well as mechanobiology as an overarching theme, respectively.

This review aims to provide a comprehensive overview of the past five years of literature on miRNAs associated with degenerative disc pathology. Particular attention will be paid to the dysregulation of miRNAs and their role in regulating the hallmarks of IVD disease, namely ECM degradation, apoptosis, and inflammation, as well as mechanobiology as an overarching theme (Figure 1).

## 2. ECM Degradation

The composition of the ECM, as well as homeostasis between ECM degradation and rebuilding are crucial for the physiological function of IVDs. NP cells play a central role in the anabolism and catabolism of the ECM, maintaining this homeostasis and compositional integrity of the tissue. More specifically, the term NP cells comprises two specific cell types: nucleopulpocytes and notochordal cells. The presence of both nucleopulpocytes and notochordal cells seems to be essential for ECM homeostasis, and the age-related loss of notochordal cells leads to an imbalance, which is connected to ECM degradation. In the pathological event of IVD degeneration, the ECM metabolism is also dysregulated due to changes in the gene expression and protein secretion of NP cells, leading to increased release of ECM degradative enzymes and decreased production of ECM structural molecules [14]. Proteolytic enzymes, such as MMPs and ADAMTS, are mainly responsible for the structural and content-related changes of the ECM [10,11]. MiRNAs, being post-transcriptional regulators, have been shown to play a crucial role in the metabolic dysregulation by regulating the gene expression of NP cells (Table 1).

Alterations in the expression of structural proteins such as type II collagen and aggrecan by miRNAs seem to occur through two main routes: either by targeting enzymes that are directly involved in ECM degradation such as MMPs [41,42,43,44], or by targeting intermediate signaling enzymes such as phosphatase and tensin homolog protein (PTEN), the IL-6/signal transducer and activator of transcription 3 (IL-6/STAT3) signaling pathway or growth differentiation factor 5 (GDF5) [45,46,47,48,49]. Concentrating first on miRNAs directly targeting MMPs, it has been confirmed that miR-93 targets and regulates MMP-3, a collagen and proteoglycan degrading enzyme. The downregulation of miR-93 observed in degenerative NP cells isolated from patients with DDD led to increased levels of MMP-3, ultimately resulting in type II collagen degradation [41]. 

Another study identified 28 differentially expressed miRNAs in NP tissue from patients suffering from DDD compared to patients with fresh lumbar fractures by next-generation sequencing (Illumina sequencing). Amongst the identified miRNAs, miR-193-3p was shown to be significantly downregulated in DDD tissues. This downregulation correlated with the grade of degeneration. Target prediction with several online prediction tools and dual luciferase reporter assay confirmed MMP-14 as a target of miR-193-3p. In vivo experiments, where DDD-related miR-193-3p downregulation was counteracted with the injection of miR-193-3p-expressing lentivirus in a DDD rat model, showed significantly increased type II collagen and aggrecan expression levels compared to the untreated control group [42].

Amongst other MMPs, the collagen degrading enzyme MMP-13 is known to be overexpressed in DDD [10]. Li et al. provided evidence that this overexpression is partially mediated through miR-27b downregulation in NP cells isolated from DDD tissue. Functional characterization confirmed that MMP-13 is a target of miR-27b [43]. Furthermore, studies have shown that miR-133a downregulation, seen in both degenerative NPs and spinal tuberculosis, resulted in the loss of type II collagen. While miR-133a negatively regulates the intracellular gelatinase MMP-9, it has also been shown to be lower expressed in degenerated IVD tissue, thus limiting the tissues’ means to control MMP-9 expression, ultimately resulting in increased MMP9-associated type II collagen degradation [44,50].

Focusing on miRNAs targeting signaling enzymes involved in ECM metabolism, miR-98 was reported to target the IL-6/STAT3 signaling pathway, a potential regulator of DDD. The downregulation of miR-98 in DDD led to increased IL-6 levels in NP tissue. In addition, reduced miR-98 levels activated the STAT3 signaling pathway by increasing protein levels of STAT3, pSTAT3, and MMP-2, thereby promoting IVD degeneration [45].

Growth differentiation factor 5 (GDF5) is known to be involved in ECM anabolism [51]. Polymorphisms of GDF5 have been associated with susceptibility to degenerative diseases such as osteoarthritis [52]. The expression of GDF5 was shown to be negatively regulated by two miRNAs in degenerated IVDs: miR-132 [46] and miR-7 [47]. miR-132 promoted ECM degradation by directly targeting GDF5 and leading to increased MMP-13 and ADAMTS4 expression through the mitogen-activated protein kinase/extracellular signal-regulated kinases (MAPK/ERK) pathway. In vivo experiments confirmed that the inhibition of miR-132 attenuated ECM degradation, making it a promising therapeutic target [46]. The role of miR-7 targeting GDF5 was studied in the context of degenerated and IL-1β-stimulated NP cells. Results demonstrated that miR-7 overexpression enhanced ECM degradation, whereas the inhibition of miR-7 reduced this effect [47].

The decreased expression of SRY-box transcription factor 9 (SOX9), a regulator of chondrogenesis, is associated with aging and degeneration of IVDs [53]. With miR-494 being upregulated in DDD and directly targeting SOX9, gain-of-function experiments showed enhanced gene expression and protein levels of ECM degradative enzymes MMP-3, MMP-13, and ADAMTS5. In parallel, loss of function experiments indicated increased type II collagen and aggrecan expression [48]. Finally, the upregulation of miR-21 in degenerated IVD tissue generates higher MMP-3 and MMP-9 expression levels by directly targeting PTEN involved in the Akt signaling pathway [54].

Taking these recent studies into account, it becomes evident that miRNAs play a key role in the metabolic dysregulation in IVD pathologies. Alterations in the miRNA expression profile of degenerative NP cells lead to the dysregulation of ECM metabolic enzymes, resulting in the degradation and compositional change of the ECM. This provides an opportunity to counteract the degenerative processes with miRNAs as therapeutic targets. For instance, a recent preclinical study evaluated the in vivo effects of injecting the inhibitor of miR-141, known to be involved in ECM degradation and apoptosis. The successful delivery of anti-miR-141 seemed to have a protective effect against DDD [55]. Another study investigated the use of high MMP levels in the ECM of degenerated IVDs for a novel two-stage delivery system of therapeutic miRNAs by encapsulating them in an outer MMP-degradable hydrogel and an inner MMP-responsive polyplex micelle for injection into IVD [56].

## 3. Apoptosis

**Figure 2:**
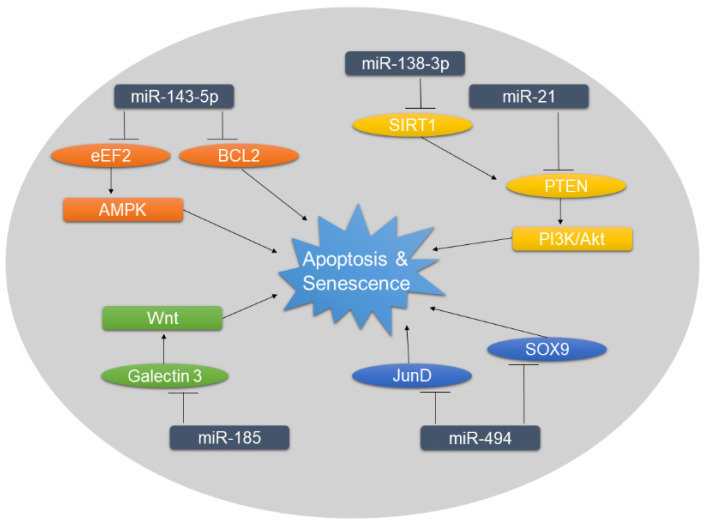
Overview of miRNAs associated with increased rates of apoptosis and senescence in DDD. Targets of negative miRNA regulation are shown in ellipses. The dysregulation of miRNAs and subsequently of their targets influence the downstream signaling, ultimately promoting apoptosis and senescence.

The degenerative processes in IVDs are associated with high rates of apoptosis and senescence, leading to decreasing cell numbers in NP tissue. Along with metabolic dysregulation, this cell loss adds to the disruption of tissue homeostasis [15,16,17,57]. Moreover, the avascular nature of the NP tissue contributes to the accumulation of cellular senescence, which is accompanied by inflammatory responses, low cell proliferation, and a catabolic phenotype, all of which take part in IVD degeneration [58]. However, mechanisms behind the increased levels of apoptosis and senescence in degenerated IVDs are, as of now, not completely understood. Changes in the expression profile of miRNA were shown to play a role in the intracellular regulatory cascade leading to apoptosis in DDD (Figure 2) and other degenerative diseases such as osteoarthritis [59]. Furthermore, miRNAs are known to be implicated in cellular senescence and senescent-associated secretory phenotype [60].

The most promising and thoroughly conducted studies investigating the role of miRNAs in IVD degeneration identified miR-185 and miR-143-5p to be associated with apoptosis. Both studies conducted in vivo experiments using DDD rat models established by needle puncture of the NP tissue [61,62]. miR-185 was found to be targeting galectin 3, a β-galactosidase-binding protein involved in apoptosis and the Wnt/β-catenin pathway [63,64]. Expression levels of galectin 3 were significantly elevated in rats with DDD compared to the healthy control group. These expression levels, as well as the activation of the Wnt/ β-catenin signaling pathway, increased even more when miR-185 was inhibited. However, transfection of miR-185 in vitro and injection of miR-185 in vivo attenuated these effects, leading to decreased rates of apoptosis [61]. miR-143-5p, on the other hand, was shown to be upregulated in degenerated NP tissue of rats. Eukaryotic elongation factor 2 (eEF2), as one of the targets of miR-143-5p, is known to be involved in apoptosis and cell proliferation, and its activation is connected with the 5’ adenosine monophosphate-activated protein kinase (AMPK) signaling pathway [65]. The dysregulation of miR-143-5p in DDD was shown to lead to the decrease of eEF2 and activation of AMPK signaling pathway, which consequently decreased type II collagen and aggrecan expression. The inhibition of miR-143-5p resulted in lower levels of apoptosis and senescence via the inactivation of AMPK in vitro [62]. In addition, the effect of miR-143 upregulation was also studied in human NP tissue isolated from patients suffering from DDD [66]. Overexpression of miR-143 was found to be proapoptotic by directly targeting and reducing intracellular B-cell lymphoma-2 (BCL2), an enzyme blocking apoptosis. Focusing also on epigenetic factors of miRNA regulation, it was shown that the promotor of miR-143 is hypo-methylated in DDD [66].

Amongst the pathways involved in apoptosis, the phosphoinositide 3-kinase (PI3K)/Akt pathway is known to play an important role especially in degenerative diseases and cancer. Importantly, this pathway is negatively regulated by PTEN [67]. Two miRNAs were found to influence apoptosis by regulating PTEN/PI3K/Akt signaling, either by directly targeting PTEN (miR-21) [49,54] or via sirtuin 1 (miR-138-3p) [68]. 

Interestingly, the upregulation of miR-494 in degenerative IVDs was reported to not only be involved in ECM degradation [48], but also in apoptosis [48,69]. Two pathways of post-transcriptional regulation of miR-494 were studied, targeting either JunD [69] or SOX9 [48]. The first study showed that TNF-α-induced apoptosis led to miR-494 upregulation, whereas the knock-down of miR-494 resulted in lower apoptosis rates via JunD upregulation [69]. A more recent study proposed that miR-494 upregulation leads to lower intracellular SOX9 levels. SOX9 is known to protect against IL-1β-induced apoptosis in other degenerative diseases, such as osteoarthritis [70]. The downregulation of SOX9 by miR-494 seen in degenerative IVDs, therefore, increased apoptosis, shedding light on mechanisms underlying increased apoptotic rates [48]. 

Other studies have shown the dysregulation of miR-660 [71], miR-145 [72], and miR-34a [73] in DDD as well as their association with increased levels of apoptosis. miR-145, targeting Adam17, repressed NP cell apoptosis in vitro, both in the presence and absence of oxidative stress [72]. On the other hand, miR-660 was significantly upregulated in DDD and after TNFα-induced apoptosis. Inhibition of miR-660 led to lower apoptosis levels and seemingly downregulated c-caspase3 and c-caspase7, both involved in apoptosis [71]. Furthermore, miR-34 was also found to be upregulated in degenerated cartilage endplate tissue, negatively regulating BCL2. Silencing of miR-34 resulted in reduced rates of apoptosis in vitro [73]. Similar results have been observed in a study investigating miR-34 in an in vitro rat osteoarthritis model [74]. However, these studies should be regarded with caution because the connection between miRNA targets and apoptosis was not addressed sufficiently and no substantial proof of the connection between these miRNAs and apoptosis was provided.

## 4. Inflammation

As one of the hallmarks of DDD, inflammation of the degenerated IVD tissue accompanied by increased release of proinflammatory cytokines is one of the key factors leading to discogenic pain. The secretion of cytokines, with TNF-α, IL-1β, and IL-6 being the most prominent ones, results in the recruitment of host immune cells (macrophages, neutrophils, and T-cells) if structural defects exist. As the inflammatory response progresses, immune cells and nociceptive nerve fibers from the dorsal root ganglion start to infiltrate the damaged IVD tissue. Once NP and immune cells start releasing neurotrophins, the nociceptive nerve fibers get stimulated and start transducing the pain sensation. Furthermore, inflammatory cytokines enhance the degenerative process by activating the expression of ECM degradative proteins and inhibiting the expression of ECM structural molecules. Additionally, these cytokines are known to contribute to cellular senescence and apoptosis [18,19,20]. The close association between miRNA and inflammation, either by being part of the cellular response to the inflammatory environment or by contributing to inflammation through dysregulating cytokines, has been described in numerous diseases [75].

A study by Dong et al. investigated the role of miR-640 in DDD and inflammation. After confirming the upregulation of miR-640 in both, DDD tissue and cells, they found that this upregulation could be caused by the inflammatory environment. Stimulation of cells with TNF-α and IL-1β led to a significant increase in miR-640 mediated through the nuclear factor-κB (NF-κB) signaling pathway. The target of miR-640 was predicted and confirmed to be the low-density lipoprotein receptor-related protein 1 (LRP1), an indirect inhibitor of NF-κB. Furthermore, they provided evidence that miR-640 is involved in the degenerative process by inducing senescence and apoptosis in NP cells, increasing expression of MMP-3 and MMP-9, and decreasing aggrecan and type II collagen [76]. miR-625-5p was also found to be induced by the NF-κB signaling pathway through the toll-like receptor 4 (TLR4), after stimulation with lipopolysaccharide (LPS). The target of miR-625-5p was confirmed to be the ECM structural protein type I collagen [77].

Evidence of the involvement of miR-194 in the inflammatory response was provided in two studies investigating two different targets. Both studies showed that miR-194 was downregulated in an inflammatory environment after treatment with LPS [78,79]. This downregulation was connected with the overexpression of the two cullin proteins CUL4A and CUL4B. The increased content of CUL4A and CUL4B was confirmed in degenerated IVD tissue, positively correlating with the degree of degeneration [79]. Furthermore, it was shown that miR-194 also targets TNF receptor-associated factor 6 (TRAF6) and that the transfection of healthy rat NP cells with miR-194 could decrease the expression of inflammatory cytokines, even after LPS treatment [78]. Interestingly, a thoroughly conducted third study showed that miR-194 together with miR-515 was upregulated in DDD tissue by inflammation [80]. These contradictory findings could arise from the fact that the two previously mentioned studies evaluated miR-194 levels by using rat NP cells [78] or human NP cell lines [79], while this study used primary NP cells isolated from human degenerated IVD tissue. Cell lines and animal models are known to show phenotypic differences compared to primary tissue, the latter being generally preferred, but only available in limited amounts. The upregulation of miR-194 and miR-515 led to the degradation of chondroitin sulfate synthase CHSY-1/2/3 in human degenerated NP cells [80]. These glycosyltransferases are responsible for the synthesis of chondroitin sulfate, a water-binding molecule which is an essential component of aggrecan [81,82,83,84]. Their results provided evidence that miRNAs act as mediators between inflammatory cytokines and ECM synthesis [80]. 

Other studies showed the inflammation-dependent dysregulation of miRNAs such as miR-149 [85], miR-181a [86], miR-146a [87], and miR-155 [88] and their involvement in intracellular signaling by targeting myeloid differentiation primary response 88 (MyD88) [85], tumor necrosis factor-related apoptosis-inducing ligand (TRAIL) [86], or transcription factor 7-like 2 [88]. Even though these studies failed to provide sufficient proof of the underlying mechanisms, the above-mentioned miRNAs might serve as potential biomarker candidates due to their strong dysregulation. For instance, preliminary screening of DDD-associated miRNAs in patient serum showed that miR-155-5p was significantly downregulated when comparing a small cohort of patients with DDD to healthy patients (*n* = 3 each) [89]. However, variations in serum miRNA content could be influenced by numerous other diseases such as obesity or diabetes, which, together with limited extracellular stability of miRNAs, poses a major limitation and challenge in finding DDD-specific circulating miRNAs.

## 5. Mechanobiology

IVDs are crucial for the flexibility of the spine, as well as for the compensation and distribution of mechanical loads. Being embedded between two vertebrae, the inner NP and outer AF tissue are connected with the vertebral bodies through cartilaginous endplates (CEP) [90]. Nonphysiological mechanical loads are one of the known factors contributing to DDD and LBP [24,25,26]. In recent decades, the impact of mechanical forces on cells and their gene expression has become of growing interest. Research clearly indicates that this connection between mechanics and biology is of complex nature, affecting several cellular processes including inflammation, ECM degradation, and apoptosis [12]. The regulation of these processes might be mediated through mechanosensitive miRNAs, which in the context of IVDs have mainly been studied in the CEP [91,92,93,94]. Degeneration of the CEP results in its calcification [95] and a decrease of thickness [96], limiting the supply of nutrients to NP tissue and thereby contributing to DDD [97].

A study by Liu et al. showed that matrix stiffness of the CEP positively correlates with the degree of DDD. Structural changes in the collagen network and increased matrix stiffness, indicating severe degeneration of CEP, promoted inorganic phosphate-induced calcification. The microRNA expression profile of CEP chondrocytes cultured on stiff matrices showed significant upregulation of miR-20a, which in turn downregulated ankyloses protein homolog (ANKH). This dysregulation of ANKH is associated with inorganic phosphate-induced calcification and with the degree of DDD in clinical samples [91]. 

Focusing on the effects of mechanical tension on miRNAs, intermittent cyclic mechanical tension (ICMT) of isolated CEP chondrocytes resulted in the significant upregulation of 21 and downregulation of 62 miRNAs. These results were validated with RT-qPCR, generating the most promising upregulated (let-7a, miR-29c, miR-142, miR-181a) and downregulated candidates (miR-H14, miR-637). Gene target prediction showed that most of these miRNAs are mainly targeting the MAPK and Wnt signaling pathways [94]. Another study identified miR-365 to be mechanosensitive after ICMT activation of CEP chondrocytes. The target of miR-365 was predicted to be histone deacetylase 4 (HDAC4) and the downregulation of miR-365 after mechanical stress led to the downregulation of type II collagen and aggrecan. This downregulation and loss of ECM structural molecules was attenuated after transfecting CEP chondrocytes with miR-365 mimics. Furthermore, the dysregulation of HDAC4 caused by the mechanosensitive miR-365 resulted in the activation of the Wnt/β-catenin signaling pathway [93]. Mechanical tension also affected the expression of miR-455-5p in endplate chondrocytes, accompanied by the dysregulation of RUNX2 [92].

In conclusion, it is evident that we currently only have very limited insight into the effects of mechanical stresses on miRNA expression and regulation. In particular, the implications of mechanosensitive miRNAs on DDD in NP and AF tissues have not yet been studied. The role of the intralamellar matrix, located between the lamellae of the AF tissue and densely packed with elastic fibers, has recently become evident in the context of micromechanical properties of the IVD [98]. Studying miRNAs in the context of disrupted intralamellar matrices and subsequent changes in the mechanical properties of the tissue would provide an interesting new direction for future research. Furthermore, focusing on the differences of beneficial and nonbeneficial mechanical loads on miRNA expression, which have already been studied in other degenerative diseases such as osteoarthritis [99], would be of big importance. This provides an opportunity for future studies to gain a better understanding of the overreaching effects of nonphysiological mechanical loads on the degenerative process. 

## 6. Conclusions

Based on the current knowledge provided herein, it is evident that pathological changes in cells of DDD tissues are associated with the dysregulation of miRNAs and their targets. Their involvement in multiple cellular processes appears to contribute to the three hallmarks of DDD: ECM degradation, apoptosis, and inflammation. A small number of studies have also provided evidence that miRNAs might be involved in the process of mechanosensing in IVD cells. However, a better understanding of the mechanisms behind the dysregulation of miRNAs and their role in DDD progression is still needed. Additionally, future studies should consider investigating the broader miRNA–mRNA network in order to gain a deeper knowledge of the regulatory pathways in DDD pathology. 

A better understanding of miRNAs in DDD provides a considerable opportunity for their use as (a) biomarkers or (b) drug targets and future therapeutics: (a) As in many other diseases, circulating miRNAs have been discussed and studied as potential biomarkers in blood for early noninvasive detection of IVD degeneration. Challenges connected with the selection of circulating miRNAs as biomarkers are the need for extensive screenings and the selection of DDD-specific dysregulated miRNAs that are not associated with other pathologies [100], the latter being one of the major limitations due to the fact that miRNAs are often linked to multiple diseases. Therefore, changes in serum miRNA levels might not only be connected to IVD degeneration, possibly making miRNA serum levels a biased reflection of the degree of degeneration. Extensive preliminary screenings have already been done in similar pathologies, such as osteoarthritis [101] or ossification of the posterior longitudinal ligament [102]. (b) As the search for novel noninvasive treatment options for DDD regeneration continues, cell therapies are amongst the most studied, ranging from the use of NP cells, chondrocytes, and mesenchymal stem cells to in vitro differentiated nucleopulpocytes and notochordal cells [33,34]. On the other hand, biological factors, such as miRNAs, provide a huge therapeutic potential in counteracting dysregulated cellular metabolism. Of course, several challenges need to be faced in the process of developing miRNA-based therapeutics. Methods of delivery, including the challenges of direct injection and miRNA vectorization, are being studied; for example, the use of injectable MMP-degradable hydrogels containing MMP-responsive polyplex micelles as a two-stage delivery system for miRNAs [56]. Other challenges that should be addressed in the future are the appropriate dosing, recognition of target cells, and timing of miRNA-based therapeutics. Moreover, potential miRNAs, as opposed to small-interfering RNAs, have to be carefully selected and meticulously studied in vitro and in vivo with regard to their nature of targeting multiple genes and their interaction network with other endogenous RNAs. 

## Figures and Tables

**Table 1:** microRNAs associated with ECM degradation in DDD and their corresponding targets. MMP: matrix metalloproteinases; IL-6/STAT3: interleukin-6/signal transducer and activator of transcription 3; GDF5: growth differentiation factor 5; SOX9: SRY-box transcription factor 9; PTEN: phosphatase and tensin homolog protein.

microRNA	Target	Reference
miR-93	MMP-3	Jing et al. (2015) [41]
miR-193-3p	MMP-14	Ji et al. (2016) [42]
miR-27b	MMP-13	Li et al. (2016) [43]
miR-133a	MMP-9	Xu et al. (2016) [44]
miR-98	IL-6/STAT3	Ji et al. (2016) [45]
miR-132	GDF5	Liu et al. (2017) [46]
miR-7	GDF5	Liu et al. (2016) [47]
miR-494	SOX9	Kang et al. (2017) [48]
miR-21	PTEN	Liu et al. (2014) [49]

## Abbreviations

3′-UTR	3′-untranslated region
ADAMTS	A disintegrin and metalloproteinases with thrombospondin motifs
AF	Annulus fibrosus
AMPK	5’ adenosine monophosphate-activated protein kinase
BCL2	B-cell lymphoma-2
CEP	Cartilaginous endplates
CHSY	Chondroitin sulfate synthase
DDD	Degenerative disc disease
ECM	Extracellular matrix
eEF2	Eukaryotic elongation factor 2
ERK	Extracellular signal-regulated kinases
GDF5	Growth differentiation factor 5
HDAC4	Histone deacetylase 4
ICMT	Intermittent cyclic mechanical tension
IL	Interleukin
IVD	Intervertebral disc
LBP	Low back pain
LPS	Lipopolysaccharide
LRP1	Low density lipoprotein receptor-related protein 1
MAPK	Mitogen-activated protein kinase
miRNA	microRNA
MMP	Matrix metalloproteinases
MyD88	Myeloid differentiation primary response 88
NP	Nucleus pulposus
PI3K	Phosphoinositide 3-kinase
PTEN	Phosphatase and tensin homolog protein
RISC	RNA-induced silencing complex
RNA	Ribonucleic acid
SOX9	SRY-box transcription factor 9
STAT3	Signal transducer and activator of transcription 3
TLR4	Toll-like receptor 4
TNFα	Tumour necrosis factor alpha
TRAF6	TNF receptor-associated factor 6
TRAIL	Tumor necrosis factor-related apoptosis-inducing ligand
